# Simplified aortic cannulation (SAC) – a useful technique for neonates with small aortas

**DOI:** 10.1186/1749-8090-1-13

**Published:** 2006-05-28

**Authors:** Christopher J Knott-Craig, Peter Pastuszko, Edward D Overholt

**Affiliations:** 1 Section of Pediatric Cardiothoracic Surgery, Children's Hospital of Oklahoma and Oklahoma University Medical Center, Oklahoma City, Oklahoma, USA; 2 Section of Cardiology, Children's Hospital of Oklahoma and Oklahoma University Medical Center, Oklahoma City, Oklahoma, USA; 3 Dept Cardiothoracic Surgery, Suite WP2230, 920 Stanton Young Blvd, Oklahoma City OK 73104, USA

## Abstract

A simplified means of arterial cannulation for cardiopulmonary bypass in small neonates and those infants with diminutive aortas, or requiring reconstruction of the ascending aorta, is presented. It involves suturing a long 3.5 mm graft to the innominate artery and inserting the arterial cannula into the end of the graft. This technique improves exposure, thereby greatly simplifying many complex repairs, and may be used for initiation of ECMO or for hybrid procedures in the postoperative period.

## Introduction

Cannulation of the diminutive ascending aorta in small neonates can be difficult. Not only can this result in narrowing of the aorta once the cannula is removed and the aortotomy closed, but the cannula itself may obstruct the aorta before and after cardiopulmonary bypass. This may contribute to unstable hemodynamics and complicate the process of weaning from bypass at the conclusion of the procedure.

In order to overcome these difficulties, we have utilized an alternative means of aortic cannulation which is both simple and reproducible. Furthermore, it has potential benefits for the patient in the perioperative period. We termed this "Simplified Aortic Cannulation" (SAC) and presented our initial experience with SAC at international meetings in September 2004 and 2005 [1]. This has become our standard aortic cannulation technique for neonates requiring reconstruction of the ascending aorta or transverse aortic arch and for those neonates and small infants with small (less than 5 mm) ascending aortas.

Between February 2000 and January 2006, 86 neonates underwent cardiac procedures utilizing SAC at the Children's Hospital of Oklahoma. These included a 1300 g preterm infant with Tetralogy of Fallot, and 2 infants with Transposition of the Great Arteries weighing less than 1800 g. Also included in this group are all neonates with Hypoplastic Left heart Syndrome (n = 57) and with Interrupted Aortic Arch complex (n = 18) operated on during this period.

## Surgical technique

**Figure 1:**
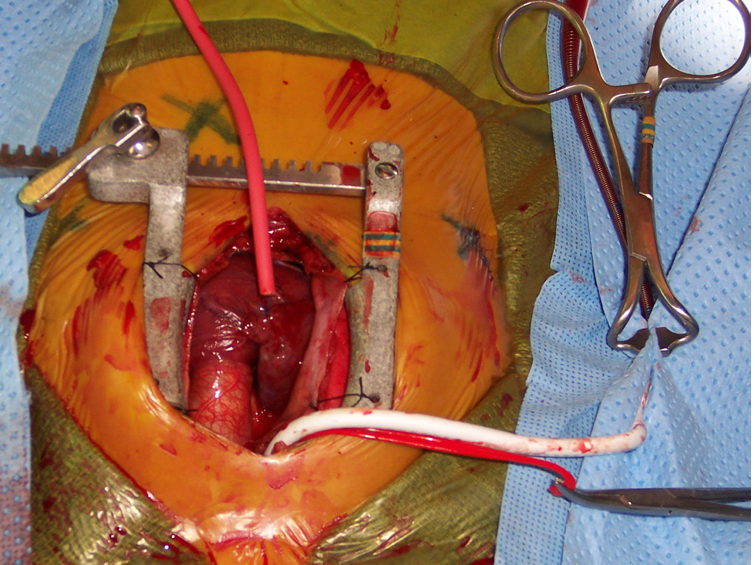
Intra-operative photo showing SAC. The 3.5 mm PTFE graft has been anastomosed to the innominate artery and is tacked away and connected to the arterial cannula.

A standard median sternotomy is performed, and the thymus gland, if large, is partially removed. The innominate artery is dissected out cephalad to the innominate vein and looped with a vessel loop. This is usually done prior to the opening of the pericardium. A Castaneda clamp is applied and a longitudinal arteriotomy is made. A 3.5 mm thin-walled PTFE graft is anastomosed end-to-side to the innominate artery using a continuous 7/0 prolene suture. The innominate artery is unclamped and the graft is flushed and clamped and left as long as possible, usually 15–20 cm. An 8Fr or 10Fr ECMO arterial cannula is inserted into the end of the graft, connected to the arterial side of the bypass circuit, and de-aired in the usual fashion. The cardioplegia line is frequently connected to the side-arm of the arterial cannula, obviating the need to place a needle in the ascending aorta for the administration of cardioplegia. The graft, being soft and flexible, easily drapes out of the operative field and is tacked to the drapes where the rigid ECMO cannula joins the graft (Figure 1). The patient is heparinized either before the graft is sutured to the innominate artery or once the anastomosis is completed. The pericardium is then opened and the right atrium or venae cavae are cannulated.

**Figure 2:**
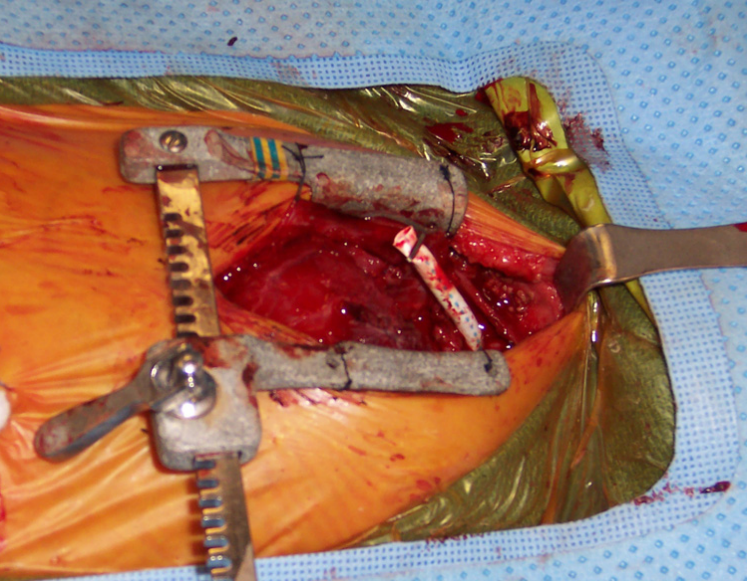
If the chest is left open, the graft is clipped about 3–4 cm long and can be re-used during the perioperative period for ECMO or hybrid procedures.

**Figure 3:**
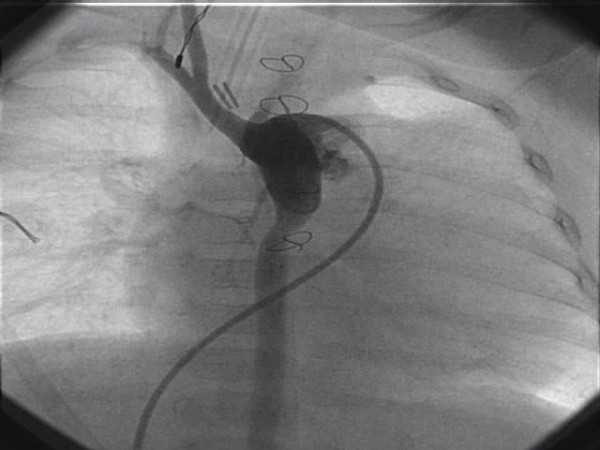
Angiogram done 4 months later, prior to second stage Norwood, showing normal contour of the innominate artery.

At the completion of the case, after the heparin has been reversed, the surgeon has two options. If a delayed sternal closure is planned, the graft is clipped and divided about 3 cm from its anastomosis to the innominate artery and tucked under the sternal edge (Figure 2). If the patient become unstable during the perioperative period, the clip can be expeditiously removed, the graft allowed to flush and an ECMO cannula reinserted into it very quickly and without potentially damaging the repaired aorta. Additionally, if a hybrid procedure is planned, this graft may give excellent access for the interventional cardiologist without injuring the small native vessels of the patient. On the other hand, if the chest is closed either in the operating room or in the ICU, a metal clip can very easily be applied to the graft close to and parallel with the innominate artery, thus avoiding sutures and injuring the vessel. Angiograms done 4 months later have shown that the innominate artery is neither distorted nor damaged by this procedure (Figure 3).

## Comments

The essential principles of SAC were described to enable regional perfusion of the brain during somatic hypothermic circulatory arrest [2-8]. We have modified the technique by leaving the graft long, and utilizing it for whole body perfusion. This allows the operating field to be uncluttered, and facilitates the repair in small neonates and other infants with small aortas. Most importantly, it enables complex surgeries on the often diminutive ascending aortas to be completed expeditiously without cumbersome cannulas obstructing the field. We have not observed any bleeding from the anastomosis between the graft and the innominate artery during or after surgery. This can be avoided by heparinizing the patient after the anastomosis is done, though we have done it both ways without problems.

Perfusion of the lower body is excellent using SAC, and the innominate or subclavian arteries have been used electively for aortic cannulation in adults with diseased aortas for many years. When the graft is later excised from the innominate artery, we place a Weck clip parallel with and flush against the artery, thus avoiding any potential blind pouch which may be a source for embolization. We do not routinely examine the innominate artery with Doppler ultrasound before discharge since we consistently observed a normal contour in the HLHS patients who routinely have a cardiac catherterization at about 3 months in preparation for the second stage.

SAC has additional potential benfits: for example, in case of emergency, it allows the surgical team to place the infant on ECMO easily and without injuring the aorta in the process, something which happens all too often. We have used this in the operating room to re-establish cardio-pulmonary bypass, and it is amazingly easy to do so provided one uses an 8Fr ECMO arterial cannula. Furthermore, it affords the interventional cardiologists and surgeons easy access to the left sided cardiac structures in the early post-operative period, for hybrid procedures. Although we have not yet needed to do this, our cardiologists believe that this would not be difficult, since the graft is sewn on at right angles to the artery, and therefore has a somewhat favourable angle of accessibility. Clearly one needs to be careful not to disrupt the suture line in the process.

SAC has changed the way we do many complicated procedures over the past 5 years. The majority of complex procedures are now done while the patient is being perfused, usually at 15 degrees rectal temperature flowing 50 ml/kg/min. Using SAC, our period of circulatory arrest for Sano-Norwood procedures, for example, rarely exceed 25 minutes even for inexperienced surgeons. Indeed, the surgery may easily be completed without circulatory arrest. This technique greatly simplifies complicated Truncus Arteriosus repairs by not having an aortic cannnula and cross-clamp in close proximity to the origin of the pulmonary arteries. The same is true for small neonates with Transposition of the Great Arteries and Aorto-Pulmonary Window. It allows patients with Interrupted Aortic Arch, especially type C, to be repaired comfortably without the need to remove and reinsert the aortic cannula close to the aortic suture line. In our experience, this has translated into improved survival for many groups of patients; for example, our hospital mortality since 2000 for all neonates with IAA complex is 5% (1/18), compared to the period prior to 2000 (32%, 16/49).

SAC appears to be a safe modification to existing techniques of aortic cannulation and may prove to be helpful when dealing with very small pre-term infants or those with unusually small ascending aortas. More frequent use of this technique could potentially further limit the indications for long periods of circulatory arrest.
